# Are measures and related symptoms of cachexia recorded as outcomes in gastrointestinal cancer chemotherapy clinical trials?

**DOI:** 10.1002/jcsm.13458

**Published:** 2024-03-27

**Authors:** Ross Valaire, Frances Garden, Valentina Razmovski‐Naumovski

**Affiliations:** ^1^ Faculty of Medicine & Health, South West Sydney Clinical Campuses University of New South Wales (UNSW) Sydney Kensington NSW Australia; ^2^ School of Medicine Western Sydney University Campbelltown NSW Australia; ^3^ Ingham Institute of Applied Medical Research Sydney NSW Australia

**Keywords:** Cachexia, Cancer, Chemotherapy, Clinical trial, Gastrointestinal, Quality‐of‐life

## Abstract

**Background:**

Cachexia is prevalent in gastrointestinal cancers and worsens patient outcomes and chemotherapy compliance. We examined to what extent registered gastrointestinal cancer chemotherapy clinical trials record measures and related symptoms of cachexia as outcomes, and whether these were associated with trial characteristics.

**Methods:**

Four public trial registries (2012–2022) were accessed for Phase II and/or III randomized controlled pancreatic, gastric, and colorectal cancer chemotherapy trial protocols. Trial outcome measures of overall survival and toxicity/side effects, and those related to cachexia [physical activity, weight/body mass index (BMI), dietary limitations, caloric intake, lean muscle mass] and symptoms (appetite loss, diarrhoea, pain, fatigue/insomnia, constipation, nausea, vomiting, and oral mucositis) were extracted, along with the number and types of performance status and patient‐reported outcomes (PROs) tools. Data were summarized descriptively. Chi‐square tests examined associations between outcomes and trial characteristics (cancer type, trial location, funding source, PROs tools, and commencement year). Statistical significance was set at *P* < 0.05.

**Results:**

We included 540 trial protocols (pancreatic (35.2%), colorectal (33.3%) and gastric (31.5%)), with most trials from Europe (44.1%). Trial lead investigator was from academia (28.3%), industry (27.6%) and government (26.3%). Allied health professional involvement (26.9%) occurred at eligibility. Adjuvant therapy in trials was mainly treatment‐related (68.1%). Additional medication included anti‐nausea (2.2%) and analgesia (0.9%). Trial protocols mostly recorded overall survival (90.4%) and toxicity (78.9%), and the symptoms appetite loss (26.1%) and diarrhoea (19.1%), with the other symptoms recorded in <10% of the trials. Reporting of physical activity (*P* = 0.001), dietary limitations (*P* = 0.002), lean muscle mass (*P* = 0.027), appetite loss (*P* < 0.001), pain (*P* = 0.001), nausea (*P* = 0.012), and oral mucositis (*P* = 0.049) varied depending cancer type. Toxicity/side effects (*P* = 0.022), physical activity (*P* < 0.001), appetite loss, nausea, and vomiting (all *P* < 0.001), diarrhoea (*P* = 0.010), pain (*P* = 0.001), fatigue/insomnia (*P* = 0.001) varied depending on the trial location. Trial funding was predominantly from private/industry (34.3%) and influenced the reporting of overall survival (*P* = 0.049), weight/BMI (*P* = 0.005), caloric intake (*P* = 0.015), and pain (*P* = 0.031). Performance status and PROs tools were mentioned in 91.2% and 46.3% of the trials, respectively. Trials that incorporated PROs tools were more likely to report cachexia related outcomes, except for overall survival, lean muscle mass, and oral mucositis. The proportion of trials measuring weight/BMI increased with trial commencement year (*P* = 0.04).

**Conclusions:**

Cachexia‐related outcomes were under‐recorded in gastrointestinal cancer chemotherapy trials. As trial patients experience a high symptom burden, cachexia‐relevant measures and symptoms should be assessed throughout the trial, and integrated with primary endpoints to support their progress.

## Introduction

Cachexia is a multifactorial phagocytic syndrome caused by cancer and is characterized by the disruption of the body's pro‐inflammatory, neuroendocrine, and nutrient metabolic homeostasis, causing appetite loss, skeletal muscle and adipose tissue wasting.^1^

Specifically, gastrointestinal cancers such as pancreatic, gastric and colorectal have the highest incidence of cachexia due to altered gut function, resulting in poor nutrient absorption and increased risk of gastrointestinal obstruction. Up to 85% of patients with gastrointestinal cancers experience cachexia (most prevalent in pancreatic cancer), which accounts for 30–50% of deaths (up to 80% in advanced pancreatic cancer).^2^ People with gastrointestinal cancers have a high rate of malnutrition accompanied with nutritional impact symptoms including nausea, stomach pain, fatigue, weight loss, and appetite loss symptoms, which are enhanced with chemotherapy.^2^, ^3^ These factors exacerbate co‐morbidities and compromises quality‐of‐life, making cancer‐specific treatment less effective.^4^

More recently, chemotherapy has been implicated in cachexia's progression via increased muscle atrophy through pro‐inflammatory cytokines.^5^ Chemotherapies that target the gastrointestinal system destroy epithelium cells^6^ and alter the microbiota, changing patients' metabolic rate.^7^ Additionally, chemotherapy causes nausea, vomiting, fatigue, mucositis, and changes in taste/smell or food preferences, contributing to difficulties in food intake, anaemia, weight loss, and appetite loss.^2^, ^8^ Thus, people undergoing chemotherapy record significantly greater weight loss (typically 4–12 kg), increasing their risk of cachexia.^8^ A retrospective study showed that 50% of patients with advanced colorectal cancer developed cachexia 24 weeks after starting chemotherapy and recorded significantly lower survival rates and increased adverse events (severe appetite loss and fatigue).^9^

A recent commentary highlighted that nutritional status is largely ignored in clinical trials despite it being a vital patient‐reported outcome (PRO) and directly related to cachexia.^10^ As chemotherapy trials focus on patient survival,^11^, ^12^ the prevalence of cachexia outcome measures and related symptoms is unknown.^12^ Thus, this prompted us to examine to what extent gastrointestinal (pancreatic, colorectal, and gastric) cancer chemotherapy trial protocols record related cachexia measures, including physical activity, weight/body mass index (BMI), dietary limitations, caloric intake and lean muscle mass, and symptoms (appetite loss, diarrhoea, constipation, nausea, vomiting, pain, fatigue/insomnia, and oral mucositis) as outcomes. We also explored whether variables such as gastrointestinal cancer type, trial location, funding source, trial commencement year, along with the utilization of PROs tools, showed discernible patterns or associations with cachexia‐related outcomes (measures and symptoms).

Given the critical impact of cachexia on cancer patients, particularly those with gastrointestinal cancers, we hypothesized that public clinical trial registries would prioritize survival outcomes, while largely neglecting cachexia‐related measures and symptoms. We also hypothesized that nutritional impact symptoms, which are vital PROs, are likely underrepresented, reflecting an oversight in addressing the multifaceted challenges posed by cachexia in gastrointestinal cancer trials.

With the high symptom burden of patients undergoing chemotherapy, the objective is to bring attention to cachexia and related outcomes, discuss the implications and highlight the need for their inclusion throughout the trial, along with primary endpoints.

## Methods

### Trial selection and eligibility

This study reviewed gastrointestinal chemotherapy clinical trials on publicly‐accessible trial registries of the United States, Australasia, the European Union/United Kingdom, and China. Other registries were excluded as trial information and English translation were unavailable. Search keywords included ‘chemotherapy’ & ‘gastric cancer &/or stomach cancer’ or ‘colorectal cancer’ or ‘pancreatic cancer’. ‘Cachexia’ was not included as cachexia trials were examined previously.^11^

Study inclusions included trial design (Phase II and/or III randomized controlled trials; open or blinded), recruitment age (≥18 years), active/recruiting status (excluding suspended, terminated, withdrawn and prematurely ended trials) and time period (1 January 2012 to 31 December 2022).

### Data extraction, items, and synthesis

We extracted data on the characteristics of the trials onto a Microsoft® Excel spreadsheet (v16.8): cancer type, trial location/type/length, year commenced, line of chemotherapy (1st, 2nd, 3rd line+ experimental), lead investigator (academia, industry, government, clinic), funding source (private/industry, academia, government, combination), adjuvant therapy, allowed medication(s), and allied health professional involvement. Along with primary trial outcome measures overall survival and toxicity/side effects, those related to cachexia, including physical activity, weight/body mass index (BMI), dietary limitations, caloric intake and lean muscle mass and symptoms including appetite loss, diarrhoea, pain, fatigue/insomnia, constipation, nausea, vomiting and oral mucositis, and the number and types of performance status and PROs tools were extracted. Outcomes were also examined within the PROs tools.

### Statistical analysis

Descriptive statistics and frequencies summarized the data. Data were converted to binary notation for analysis in IBM® SPSS® Statistics (v.26, 2019, USA). Pearson's chi‐square/Fisher's exact tests examined associations between the trial outcomes and: cancer type, trial location, funding source, and PROs tools. Chi‐square test of trend assessed associations between the trial length and number of trials; the outcomes and trial year. *P* < 0.05 was considered statistically significant.

## Results

### Search and screening process of registered trials

**Figure 1:**
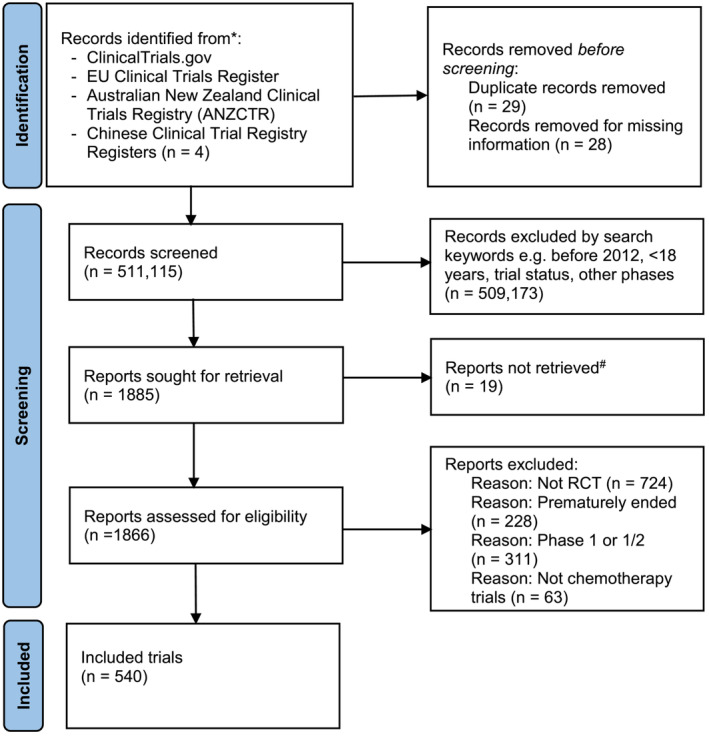
Flow chart for study selection using the Preferred Reporting Items for Systematic Reviews and Meta‐analyses flow diagram (Moher, D., Altman, D.G., Liberati, *A*. & Tetzlaff, *J*. PRISMA statement. Brittish Medical Journal 22, 128 (2011)). ^#^Could not be found prior to analysis. *https://clinicaltrials.gov/; https://www.clinicaltrialsregister.eu/; https://www.anzctr.org.au/; https://www.chictr.org.cn/indexEN.html.

From 1885 trial protocols, 1345 were excluded due to duplication, not RCT, prematurely ended, Phase I or I/II trials and not chemotherapy trials, resulting in 540 trials included in the analysis (Figure 1).

### Trial characteristics

The main cancer type of the trials were evenly distributed among pancreatic (35.2%, *n* = 190), colorectal (33.3%, *n* = 180), and gastric (31.5%, *n* = 170) (Table 1). Most trials were from Europe (44.1%, *n* = 238), followed by Asia (33.3%, *n* = 180), North America (20.7%, *n* = 112), and Australasia (1.9%, *n* = 10). The majority of trials were open/unblinded (76.9%). The distribution of trials per year increased from 5.4% (*n* = 29) in 2012 to around 10–13% between 2018–2022 (*n* = 57–69) and 56.1% of trials ran for more than 2 years. Chemotherapy use in trials was predominantly 1st line (78%, *n* = 421), compared with 2nd line (13.3%, *n* = 72), and 3rd line+ experimental (8.7%, *n* = 47). The affiliation of the trial lead investigator was evenly proportioned between academia (28.3%, *n* = 153), industry (27.6%; *n* = 149), and government (26.3%, *n* = 142). Trials were predominantly funded by private/industry (34.3%; *n* = 185), followed by government (24.8%; *n* = 134) and academia (23.9%; *n* = 129) sources. Adjuvant therapies reporting occurred in 76.5% (*n* = 391) of trials and were mainly adjuvant cancer treatments (68.1%; *n* = 368), followed by appetite stimulants (4.1%; *n* = 22). Additional medications allowed in trials were anti‐nausea (2.2%; *n* = 12) and analgesia (0.9%; *n* = 5). Allied health professionals were involved in 26.9% (*n* = 145) of trials and were required before the trial (25.2%; *n* = 136). Of these, a psychologist was the prominent allied health professional (93.1%; *n* = 135). Only one trial PROs tool was used in 32.4% (*n* = 175) of the trials. With 91.2% of trial protocols including a performance scale tool (three noted), the Eastern Cooperative Oncology Group (ECOG) Performance Status Scale was mostly cited (86.5%; *n* = 467) (Table 2).

**Table 1:** Characteristics of the included gastrointestinal clinical trials (*n* = 540)

Characteristics of clinical trials	Number of trials
	*n* (%)
Cancer type	
Pancreatic	190 (35.2)
Colorectal	180 (33.3)
Gastric	170 (31.5)
Trial location	
Europe	238 (44.1)
Asia	180 (33.3)
North America	112 (20.7)
Australasia	10 (1.9)
Trial type	
Open	415 (76.9)
Blinded	125 (23.1)
Year commenced (actual or estimated^a^)	
2012	29 (5.4)
2013	34 (6.3)
2014	47 (8.7)
2015	45 (8.3)
2016	40 (7.4)
2017	46 (8.5)
2018	62 (11.5)
2019	57 (10.6)
2020	57 (10.6)
2021	69 (12.8)
2022	54 (10.0)
Trial length*	
<1 month	9 (1.7)
1–6 months	34 (6.3)
7–<12 months	46 (8.5)
1–2 years	105 (19.4)
2+ years	303 (56.1)
Unknown^a^	43 (8.0)
Line of chemotherapy	
1st line	421 (78.0)
2nd line	72 (13.3)
3rd line+ experimental	47 (8.7)
Lead investigator	
Academia	153 (28.3)
Industry	149 (27.6)
Government	142 (26.3)
Clinic	96 (17.8)
Funding source	
Private/Industry	185 (34.3)
Government	134 (24.8)
Academia	129 (23.9)
Academia and Government	92 (17.0)
Adjuvant therapy	
Cancer treatment	368 (68.1)
Appetite stimulants^b^	22 (4.1)
Other^c^	23 (4.3)
None	127 (23.5)
Allowed medication	
None stated	494 (91.5)
Anti‐nausea	12 (2.2)
Analgesic	5 (0.9)
Other^d^	29 (5.4)
Allied health professional involvement^e^	
No	395 (73.1)
Yes	145 (26.9)
If ‘yes’ to allied health: Psychologist	135 (25.0)
Dietitian	7 (1.3)
Exercise physiologist	3 (0.6)
Allied health professional (not stated)	1 (0.2)
Timing of allied health professional involvement	
Before trial	136 (25.2)
During trial	8 (1.5)
After trial	1 (0.2)
Number of patient‐reported outcomes tools used**	
1	175 (32.4%)
2 or more	57 (10.6%)

^a^ Some trials gave registration dates, not dates trial commenced.

^b^ For example, dronabinol, anamorelin, and fish oil. Of the 22 trials, adjunct therapies were the primary focus in 11 trials, 5 were secondary and 6 were a combined focus on both chemotherapy + adjuvant therapy; 14 trials used a quality‐of‐life tool. One trial utilized an assessment tools specific to the adjunct therapy.

^c^ Adjuvant treatment not used to treat cancer or cachexia.

^d^ Medication recommended that was not adjuvant therapy or for cachexia symptoms, for example, Modafinil.

^e^ Excluding medical doctors and nurses. Percentages add to over 100.

* *P* = 0.038 association between number of trials and trial length using chi‐square test of trend.

** *P* = 0.039 association between assessment tools and allied health professionals using chi‐square.

**Table 2:** Frequency of performance status and patient‐reported outcomes tools in the included gastrointestinal clinical trials (*n* = 540)

Performance status tool	Frequency (%)	
Eastern Cooperative Oncology Group Performance Status (ECOG)	467 (86.5)	
Karnofsky Performance Status	23 (4.3)	
Zubrod Performance Status	2 (0.4)	
No tool	48 (8.9)	
Patient‐reported outcomes tool^a^		Outcomes examined (number)^b^
European Organisation for Research and Treatment of Cancer 30 (EORTC QLQ C‐30)	138 (25.6)	Physical activity (6), sleep/fatigue (3), pain (2), nausea (1), vomiting (1), diarrhoea (1), constipation (1), appetite loss (1)
European Organisation for Research and Treatment of Stomach Cancer 22 (EORTC QLQ‐STO22)	37 (6.9)	Dietary limitations (3), weight (1), pain (1)
European Organisation for Research and Treatment of Pancreatic Cancer 26 (EORTC QLQ‐PAN26)	29 (5.4)	Pain (2), side effects (1), physical activity (1), weight (1), dietary limitations (1), caloric intake (1)
EuroQol Group 5 Dimensions 5 Dimension 5 Level (EQ‐5D‐5L)	28 (5.2)	Physical activity (3), pain (1)
Functional Assessment of Cancer Therapy – General (FACT‐G)	5 (0.9)	Physical activity (3), pain (1), side effects (1), sleep (1), nausea (1)
Hospital Anxiety and Depression Scale (HADS score)	3 (0.6)	Physical activity (1)
Short Form 36 (SF‐36)	3 (0.6)	Physical activity (19), pain (2), fatigue (2)
Short Form 12 (SF‐12)	2 (0.4)	Physical activity (7), pain (1)
European Organisation for Research and Treatment of Cancer in Elderly 14 (EORTC QLQ‐ELD 14)	2 (0.4)	Physical activity (3), pain (1)
European Organisation for Research and Treatment of Chemotherapy Induced Peripheral Neuropathy (EORTC QLQ‐CIPN20)	1 (0.2)	Physical activity (3), pain (2),
Obesity and Weight Loss Quality of Life (OWL‐QoL‐17)^c^	1 (0.2)	Weight (12)^c^
Worry of Cancer Progression Scale (WOPS)	1 (0.2)	0
Not specified^d^	43 (8.0)	–
No tool	305 (56.5)	

^a^ Some trials used more than one tool.

^b^ Assessment tools and questions were reviewed based on the relevant outcomes studied.

^c^ Questions that could be related to weight loss.

^d^ Listed as quality‐of‐life tool.

### Trial outcomes (measures and symptoms reported)

The most common trial measures reported were overall survival and toxicity/side effects (90.4%, *n* = 488 and 78.9%, *n* = 426), respectively, followed by physical activity (28.9%, *n* = 156), weight/BMI (11.5%, *n* = 62), and dietary limitations (10.0%, *n* = 54). The most recorded symptom in the trials was appetite loss (26.1%, *n* = 141), followed by diarrhoea (19.1%, *n* = 103), with the other measures/symptoms recorded in <10% of trials (Table 3).

**Table 3:** Outcomes recorded in the included gastrointestinal clinical trials (*n* = 540) and associations between outcomes recorded and: Cancer type, trial location, funding source and patient‐reported outcomes (PRO) tools

Variable outcome	Yes/No	Number (*n* = 540)	Cancer type				Trial location					Funding source					PROs tool		
			Pancreatic (*n* = 190)	Gastric (*n* = 170)	Colo‐rectal (*n* = 180)	*P*‐value	Europe (*n* = 238)	Asia (*n* = 180)	North America (*n* = 112)	Austral‐Asia (*n* = 10)	*P*‐value	Private/industry‐funded (*n* = 185)	Academia (*n* = 134)	Government (*n* = 129)	Academia + government (*n* = 92)	*P*‐value	Yes (*n* = 235)	No (*n* = 305)	*P*‐value
**Measures**		***n* (%)**	***n* (%)**	***n* (%)**	***n* (%)**		***n* (%)**	***n* (%)**	***n* (%)**	***n* (%)**		***n* (%)**	***n* (%)**	***n* (%)**	***n* (%)**		***n* (%)**	***n* (%)**	
Overall survival	Yes	488 (90.4)	176 (92.6)	156 (91.8)	156 (86.7)	0.115	218 (91.6)	159 (88.3)	102 (91.1)	9 (90.0)	0.721	176 (95.1)	116 (86.6)	115 (89.1)	81 (88.0)	**0.049** *	219 (93.2)	269 (88.2)	0.051
	No	52 (9.6)	14 (7.4)	14 (8.2)	24 (13.3)		20 (8.4)	21 (11.7)	10 (8.9)	1 (10.0)		9 (4.9)	18 (13.4)	14 (10.9)	11 (12.0)		16 (6.8)	36 (11.8)	
Toxicity/side effects	Yes	426 (78.9)	146 (76.8)	141 (82.9)	139 (77.2)	0.293	199 (83.6)	129 (71.7)	89 (79.5)	9 (90.0)	**0.022** *	157 (84.9)	99 (73.9)	101 (78.3)	71 (77.2)	0.182	213 (90.6)	213 (69.8)	**<0.001** *
	No	114 (21.1)	44 (23.2)	29 (17.1)	41 (22.8)		39 (16.4)	51 (28.3)	23 (20.5)	1 (10.0)		28 (15.1)	35 (26.1)	28 (21.7)	21 (22.8)		22 (9.4)	92 (30.2)	
Physical activity	Yes	156 (28.9)	72 (37.9)	47 (27.6)	37 (20.6)	**0.001** *	89 (37.4)	29 (16.1)	33 (29.5)	5 (50.0)	**<0.001** *	63 (34.1)	29 (21.6)	35 (27.1)	33 (35.9)	0.085	153 (65.1)	3 (1.0)	**<0.001** *
	No	384 (71.1)	118 (62.1)	123 (72.4)	143 (79.4)		149 (62.6)	115 (83.9)	79 (70.5)	5 (50.0)		122 (65.9)	105 (78.4)	94 (72.9)	59 (64.1)		82 (34.9)	302 (99.0)	
Weight/BMI	Yes	62 (11.5)	29 (15.3)	19 (11.2)	14 (7.8)	0.077	28 (11.5)	16 (8.9)	16 (14.3)	2 (20.0)	0.425	26 (14.1)	9 (6.7)	9 (7.0)	18 (19.6)	**0.005** *	37 (15.7)	25 (8.2)	**0.006** *
	No	478 (88.5)	161 (84.7)	151 (88.8)	166 (92.2)		210 (88.5)	164 (91.1)	96 (85.7)	8 (80.0)		159 (85.9)	125 (93.3)	120 (93.0)	74 (80.4)		198 (84.3)	280 (91.8)	
Dietary limitations	Yes	54 (10.0)	21 (11.1)	26 (15.3)	7 (3.9)	**0.002** *	27 (11.3)	10 (5.6)	15 (13.4)	2 (20.0)	0.073	26 (14.1)	8 (6.0)	10 (7.8)	10 (10.9)	0.086	52 (22.1)	2 (0.7)	**<0.001** *
	No	486 (90.0)	169 (88.9)	144 (84.7)	173 (96.1)		211 (88.7)	170 (94.4)	97 (86.6)	8 (80.0)		159 (85.9)	126 (94.0)	119 (92.2)	82 (89.1)		183 (77.9)	303 (99.3)	
Caloric intake	Yes	13 (2.4)	7 (3.7)	3 (1.8)	3 (1.7)	0.61	8 (3.4)	1 (0.6)	4 (3.6)	0 (0)	0.218	2 (1.1)	2 (1.5)	8 (6.2)	1 (1.1)	**0.015** *	10 (4.3)	3 (1)	**0.014** *
	No	527 (97.6)	183 (96.3)	167 (98.2)	177 (98.3)		230 (96.6)	179 (99.4)	108 (96.4)	10 (100)		183 (98.9)	132 (98.5)	121 (93.8)	91 (98.9)		225 (95.7)	302 (99)	
Lean muscle mass	Yes	13 (2.4)	9 (4.7)	1 (0.6)	3 (1.7)	**0.027** *	7 (2.9)	3 (1.7)	3 (2.7)	0 (0)	0.803	3 (1.6)	7 (5.2)	2 (1.6)	1 (1.1)	0.107	9 (3.8)	4 (1.3)	0.058
	No	527 (97.6)	181 (95.3)	169 (99.4)	177 (98.3)		231 (97.1)	177 (98.3)	109 (97.3)	10 (100)		182 (98.4)	127 (94.8)	127 (98.4)	91 (98.9)		226 (96.2)	301 (98.7)	
**Symptoms**																			
Appetite loss	Yes	141 (26.1)	67 (35.3)	45 (26.5)	29 (16.1)	**<0.001** *	85 (35.7)	25 (13.9)	28 (25.0)	3 (30.0)	**<0.001** *	58 (31.4)	24 (17.9)	33 (25.6)	30 (32.6)	0.055	139 (59.1)	2 (0.7)	**<0.001** *
	No	399 (73.9)	123 (64.7)	125 (73.5)	151 (83.9)		153 (64.3)	155 (86.1)	84 (75.0)	7 (70.0)		127 (68.6)	110 (82.1)	96 (74.4)	62 (67.4)		96 (40.9)	303 (99.3)	
Diarrhoea	Yes	103 (19.1)	37 (19.5)	33 (19.4)	33 (18.3)	0.953	58 (24.3)	23 (12.8)	22 (19.6)	0 (0)	**0.010** *	36 (19.5)	20 (14.9)	28 (21.7)	18 (19.6)	0.534	55 (23.4)	48 (15.7)	**0.025** *
	No	437 (80.9)	153 (80.5)	137 (80.6)	147 (81.7)		180 (75.6)	157 (87.2)	90 (80.4)	10 (100)		149 (80.5)	114 (85.1)	101 (78.3)	74 (80.4)		180 (76.6)	257 (84.3)	
Pain	Yes	50 (9.3)	29 (15.3)	13 (7.6)	8 (4.4)	**0.001** *	23 (9.7)	6 (3.3)	18 (16.1)	3 (30)	**0.001** *	22 (11.9)	7 (5.2)	8 (6.2)	14 (15.2)	**0.031** *	43 (18.3)	7 (2.3)	**<0.001** *
	No	490 (90.7)	161 (84.7)	157 (92.4)	172 (95.6)		215 (90.3)	174 (96.7)	94 (83.9)	7 (80)		163 (88.1)	127 (94.8)	121 (93.8)	78 (84.8)		192 (81.7)	298 (97.7)	
Fatigue/insomnia	Yes	36 (6.7)	19 (10.0)	9 (5.3)	8 (4.4)	0.069	13 (5.5)	6 (3.3)	17 (15.2)	0 (0)	**0.001** *	15 (8.1)	6 (4.5)	7 (5.4)	9 (9.8)	0.387	30 (12.8)	6 (2.0)	**<0.001** *
	No	504 (93.3)	171 (90.0)	161 (94.7)	172 (95.6)		225 (94.5)	174 (96.7)	95 (84.8)	10 (100)		170 (91.9)	128 (95.5)	122 (94.6)	83 (90.2)		205 (87.2)	299 (98.0)	
Constipation	Yes	35 (6.5)	14 (7.4)	11 (6.5)	10 (5.6)	0.778	16 (6.7)	7 (3.9)	12 (10.7)	0 (0)	0.111	15 (8.1)	6 (4.5)	5 (3.9)	9 (9.8)	0.188	22 (9.4)	13 (4.3)	**0.017** *
	No	505 (93.5)	176 (92.6)	159 (93.5)	170 (94.4)		222 (93.3)	173 (96.1)	900 (89.3)	10 (100)		170 (91.9)	128 (95.5)	124 (96.1)	83 (90.2)		213 (90.6)	292 (95.7)	
Nausea	Yes	32 (5.9)	19 (10.0)	7 (4.1)	6 (3.3)	**0.012** *	10 (4.2)	5 (2.8)	16 (14.3)	1 (10.0)	**<0.001** *	12 (6.5)	7 (5.2)	8 (6.2)	6 (6.5)	0.978	25 (10.6)	7 (2.3)	**<0.001** *
	No	508 (94.1)	171 (90.0)	163 (95.9)	174 (96.7)		228 (95.8)	175 (97.2)	96 (85.7)	9 (90.0)		173 (93.5)	127 (94.8)	121 (93.8)	86 (93.5)		210 (89.4)	298 (97.7)	
Vomiting	Yes	32 (5.9)	16 (8.4)	9 (5.3)	7 (3.9)	0.167	8 (3.4)	6 (3.3)	17 (15.2)	1 (10.0)	**<0.001** *	12 (6.5)	7 (5.2)	5 (3.9)	9 (9.8)	0.315	22 (9.4)	10 (3.3)	**0.003** *
	No	508 (94.2)	174 (91.6)	161 (94.7)	173 (96.1)		230 (96.6)	174 (96.7)	95 (84.8)	9 (90.0)		173 (93.5)	127 (94.8)	124 (96.1)	83 (90.2)		213 (90.6)	295 (96.7)	
Oral mucositis	Yes	3 (0.6)	0 (0)	0 (0)	3 (1.7)	**0.049** *	1 (0.4)	1 (0.6)	1 (0.9)	0 (0)	0.947	2 (1.1)	1 (0.7)	0 (0.0)	0 (0.0)	0.523	2 (0.9)	1 (0.3)	0.417
	No	537 (99.4)	190 (100)	170 (100)	177 (98.3)		237 (99.6)	179 (99.4)	111 (99.1)	10 (100)		183 (98.9)	133 (99.3)	129 (100.0)	92 (100.0)		233 (99.1)	304 (99.7)	

*P*‐value is from a chi‐squared test or Fisher's exact test if cell sizes were small.

BMI, body mass index.

* *P <* 0.05 (bolded) means there is an association between the outcomes and variables.

### Associations between trial outcomes and trial characteristics

#### Trial outcomes across cancer type

The reporting of trial outcome measures physical activity (*P* = 0.001), dietary limitations (*P* = 0.002), lean muscle mass (*P* = 0.027), and symptoms appetite loss (*P* < 0.001), pain (*P* < 0.001), nausea (*P* = 0.012) and oral mucositis (*P* = 0.049), varied significantly across cancer type (Table 3). Caloric intake, lean muscle mass, constipation, vomiting and oral mucositis were recorded in <10% of trials for the three cancer types. Generally, pancreatic trial protocols recorded cachexia‐related outcomes more frequently than gastric and colorectal trial protocols.

#### Trial outcomes across trial location

Trial outcome measures toxicity/side effects (*P* = 0.022), physical activity; and symptoms appetite loss, nausea, vomiting (all *P* < 0.001), fatigue/insomnia, pain (both *P* = 0.001) and diarrhoea (*P* = 0.010) significantly varied with the trial location (Table 3). Generally, trial protocols from Asia recorded less cachexia‐related outcomes compared with other regions. Caloric intake, lean muscle mass and oral mucositis were recorded in <10% of trials for the studied locations.

#### Trial outcomes across funding source

Trial outcome measures overall survival (*P* = 0.049), weight/BMI (*P* = 0.005), caloric intake (*P* = 0.015), and the symptom pain (*P* = 0.031) varied significantly with funding source (Table 3). Trials funded by private/industry measured outcomes overall survival, toxicity/side effects, dietary limitations, and oral mucositis more frequently. Caloric intake, lean muscle mass, fatigue/insomnia, constipation, nausea, vomiting, and oral mucositis were recorded in <10% of trials for all funding sources.

#### Trial outcomes and patient‐reported outcomes tools

Trials that used PROs tools were more likely to report the cachexia‐related outcomes except overall survival, lean muscle mass and oral mucositis (*P* > 0.05; Table 3). Twelve specific PROs tools were cited in 46.3% of the trials (*n* = 250), of which the European Organisation for Research and Treatment of Cancer Quality of Life Questionnaire (EORTC QLQ‐C30) was the most cited tool (55.2%; *n* = 138). Physical activity was the most measured outcome within the 12‐noted PROs tools, followed by pain (Table 2).

### Changes in outcomes reported in trials by trial commencement year

The proportion of cachexia‐related outcomes reported in all trials by year is shown in Table 4. The percentage of trials recording weight/BMI significantly increased with year (*P* = 0.004). There was no significant linear trend for any other outcome reported in this study.

**Table 4:** Percentage of outcomes reported in the included gastrointestinal clinical trials by commencement year

Year	2012	2013	2014	2015	2016	2017	2018	2019	2020	2021	2022	*P*‐value
**Measures (as %)**												
Overall survival	75.9	88.2	93.6	91.1	95.0	91.3	98.4	89.5	89.5	88.4	87.0	0.151
Toxicity/Side effects	79.3	76.5	83.0	77.8	90	76.1	77.4	86.0	63.2	79.7	81.5	0.159
Physical activity	17.2	26.5	25.5	22.2	30.0	34.8	30.6	40.4	28.1	30.4	24.1	0.581
Weight/BMI	0	11.8	8.5	8.9	17.5	4.3	1.6	21.1	12.3	13.0	19.4	**0.004** *
Dietary limitations	6.9	5.9	10.6	11.1	7.5	4.3	11.3	12.3	8.8	17.4	7.4	0.621
Caloric intake	0	0	2.1	6.7	2.5	2.2	0	3.5	5.3	2.9	0	0.418
Lean muscle mass	3.4	2.9	4.3	2.2	5	0	0	5.3	5.3	0	0	0.308
**Symptoms (as %)**												
Appetite loss	10.3	23.5	25.5	22.2	30	32.6	30.6	35.1	22.8	29.0	16.7	0.306
Diarrhoea	17.2	11.8	14.9	28.9	25.0	21.7	29.0	19.3	17.5	13.0	11.1	0.191
Pain	3.4	2.9	8.5	15.6	10.0	10.9	8.1	14.0	7.0	7.2	11.1	0.663
Fatigue/insomina	3.4	2.9	2.1	6.7	5.0	6.5	6.5	10.5	14.0	5.8	5.6	0.484
Constipation	3.4	0	2.1	13.3	7.5	8.7	6.5	7.0	12.3	2.9	5.6	0.228
Nausea	0	2.9	2.1	6.7	12.5	6.5	3.2	7.0	10.5	7.2	3.7	0.393
Vomiting	3.4	2.9	2.1	6.7	10	4.3	4.8	8.8	10.5	4.3	5.6	0.729
Oral mucositis	3.4	0	0	0	0	0	0	0	0	1.4	1.9	0.513

BMI, body mass index.

* *P* < 0.05 considered significant using chi‐square test of trend.

## Discussion

Cachexia is frequently observed in gastrointestinal cancers and is associated with poorer outcomes during chemotherapy.^13^ Our study showed that measures (physical activity, weight/BMI, dietary limitations, caloric intake, lean muscle mass) and related symptoms (appetite loss, diarrhoea, pain, fatigue/insomnia, constipation, nausea, vomiting, and oral mucositis) of cachexia were under‐recorded as outcomes in pancreatic, gastric and colorectal cancer chemotherapy clinical trials.

Chemotherapy's primary aim is to improve survival. As expected, two predominant outcomes were recorded in the trials: (1) overall survival which is the primary objective endpoint for evaluating drugs and the ‘gold standard’ determining patients' care^12^ and (2) drug toxicity which impacts the individual's quality‐of‐life and physical health, with over 86% of patients report at least one side effect of chemotherapy.^14^ However, the real‐world application of trialled drugs has shown decreased overall survival by 5.2 months across all systemic therapies, and 14% higher average toxicity compared with the results of trials.^15^ These two outcomes have their limitations given that (1) cure is less likely with metastasis^12^ and (2) weight loss is generally not measured pre‐treatment (<20% weight loss is considered acceptable to tolerate treatment side effects) and reduces the effectiveness and increases the toxicity of chemotherapy, with 30% or more weight loss considered incompatible with survival.^10^ Coupled with disease and treatment‐related morbidity, cachexia and related symptom experience could be contributing to this decline, inevitably leading to missing data in chemotherapy trials which could impact the final outcome results of the trial.^16^

Nutrition interventions and physical activity have positive effects on muscle mass and physical functioning, and are recommended during cancer treatment.^17^ This study revealed differences in these recorded outcomes among various trials of gastrointestinal cancer types. Nutritional impact symptoms such as appetite loss, pain, nausea, and oral mucositis are common in gastrointestinal cancers^18^ and exhibited variations in this study. These symptoms reduce food intake, resulting in weight loss and thus, cachexia worsens with ongoing treatment if it is not addressed.^4^, ^19^ Pancreatic cancer trials recorded pain and nausea more frequently, possibly due to the high prevalence of these symptoms in people with advanced pancreatic cancer.^20^ Overall, the findings underscore the importance of considering these outcomes in the three gastrointestinal cancer types.

The geographical location of the trials had a notable effect on the documentation of outcomes, particularly toxicity, physical activity, and various symptoms. Trials conducted in Asia recorded these outcomes less frequently, potentially reflecting cultural disparities. A previous study noted that people with cancer in the Asian region appear to face more significant perceived barriers to pain management compared with their Western counterparts, offering a potential explanation for the observed differences in outcome recording frequency.^21^

In our study, privately/industry‐funded trials was associated with the rates of assessing overall survival, most likely being influenced by the end goal of regulatory approval of the drug and expanded market share.^22^ Except for caloric intake, lean muscle mass and diarrhoea, private/industry funding, or government + academia funding recorded outcomes more frequently most likely due to more funding available from these two sources.

Overall, the outcomes caloric intake, lean muscle mass, and oral mucositis were measured least. As chemotherapy dose calculations do not account for body composition, this needs to be urgently addressed in trials as decreased lean muscle mass is an important risk factor for cachexia and is directly associated with increased chemotoxicity and gastrointestinal cancer toxicity, manifesting as diarrhoea, nausea and appetite loss.^23^ Ideally, if the mucosal surface is intact, this could pre‐empt malnutrition risks and lean muscle mass loss. With around 80% of patients experiencing oral mucositis after high‐dose chemotherapy, this common problem needs urgent attention.^24^

This study looked at whether the trial year influenced the trial outcomes reported. It was revealed that weight/BMI was recorded more often in recent years. This upward trend potentially followed Fearon et al.'s definition^25^ of cachexia based on >5% weight loss or BMI < 20 kg/m^2^ but most likely reflects dosing requirements. Studies suggest that weight loss should be considered when screening patients and stratification for clinical trials to provide valid trial results.^26^ An earlier study showed weight loss was associated with lower quality‐of‐life, less likelihood to respond to chemotherapy and poor survival in advanced gastrointestinal cancers.^27^ In a retrospective cohort study of patients with advanced gastric cancer, weight loss was observed in more than 50% of the patients within 12 weeks after starting chemotherapy and was related to adverse events or reduced survival. It was suggested that monitoring weight during chemotherapy could predict adverse events such as appetite loss and fatigue.^4^

It was found that the actual screening of cachexia or cachexia‐related symptoms before commencing the trial is very low. As expected, almost all protocols used a performance status tool, with ECOG as the most often used. This and other eligibility inclusion criteria provide prognostic power and a patient's functional status as a predictor of survival from a clinician's and trials's perspective but lack holistic screening for cachexia.^28^, ^29^ Thus, a cachexia and ECOG score may identify patients at eligibility with an increased risk for developing severe toxicity events during chemotherapy treatment for gastrointestinal cancer.^23^

In addition, our study showed that PROs tools were mentioned infrequently, and there was an association between (the lack of) tools and health professionals. The main PROs tool found in this study was the EORTC QLQ‐C30 (and related iterations) and this focusses on a variety of health domains including physical, emotional, cognitive and social functioning to assess quality‐of‐life. This tool has been shown to have a strong prognostic value for overall survival in certain cancers such as colon and rectal.^30^ However, the EORTC fails to accurately assess all domains for an individual's risk for cachexia and has not been validated to assess patients for cachexia outcomes, and its responsiveness to changes in patients with cachexia has not been determined.^31^

When PROs tools were used, this was significantly associated with measuring most of the listed outcomes. However, physical activity and pain were inadvertently captured through general PROs tools, and very few of the listed outcomes were actually measured. A review highlighted that PROs and symptoms such as appetite loss, fatigue, and pain predicted overall survival in colorectal cancer, and these need to be incorporated in future gastrointestinal trials.^32^

Our study revealed important insights and highlights concerns and challenges for assessing cachexia in clinical trials. Although PROs are important from a clinical data collection perspective, these outcomes were not shared with the investigators, typically analysed post‐trial, influencing future trial design, rather than outcomes that the clinical team could use to monitor the participants during the trial. This reflects our finding that allied health professional involvement in the protocols was missing or unknown and consisted mainly of psychologists who examined eligibility for the trial. As more than 50% of the trials were longer than 2 years, this dearth of specialized support during the trial provides ample opportunity for cachexia to progress and impact an individual's dose–response and quality‐of‐life.^33^ This relates to our finding that nearly all trials did not mention medication(s) that alleviated or managed cachexia‐related symptoms which likely reflects avoiding potential drug–drug interactions during the trial (which can lead to suboptimal therapy), or symptoms experienced being unique to the patient.^34^, ^35^ If hospital stays are short during the trial period, this places a significant burden on patients to manage symptoms at home and thus, may not be monitored frequently.^19^ Ultimately, this indicates that there is no support for patients who are cachectic before, during and after the trial. We emphasize the need for more targeted assessments incorporating cachexia measures (such as nutritional status) and symptoms during the trial which would enhance the clinical endpoints used for determining the benefits and toxicity of treatment, encourage better patient‐health professional interaction to alleviate symptoms during the trial and support the on time/on dose chemotherapy treatment.^10^, ^30^, ^33^, ^36^ A recent study in a Japanese population with advanced cancer showed that the Functional Assessment of Anorexia/Cachexia Therapy Anorexia Cachexia Subscale was sensitive in identifying patients at risk of deterioration and is recommended as a quality‐of‐life tool in future clinical trials.^37^ This will provide insight into the value of patient‐reported assessments and an opportunity to examine their applicability to cachexia.^38^

We recognize that our study's findings are constrained by the variables included in the analysis, and alternative patterns may have emerged with the inclusion of additional variables, such as handgrip strength and lean body mass. Moreover, the potential loss of transferred data during translation to English, particularly with trials originating from Asia, is a notable limitation. We also acknowledge that we did not verify whether corresponding published trial results offered more trial information on outcome measures. The scarcity of trials from the Australasian region further emphasizes the need for cautious interpretation of our analysis. Additionally, our study specifically focuses on gastrointestinal cancers, and extrapolating these results to other cancer types, such as lung cancer with prevalent cachexia, or those with a high public profile, such as breast cancer, should be done with awareness of this limitation. The numerous tests of association performed in this study to assess the relationship between the trial outcomes and trial characteristics may have inflated Type‐I error rates, and as such, cautious interpretation of these secondary analyses in isolation should be made.

## Conclusion

Our study shows the under‐recording of cachexia measures and symptoms as outcomes in gastrointestinal cancer chemotherapy clinical trials in the reviewed period. It is recommended that outcomes, especially weight loss and nutritional status, are included in eligibility inclusion criteria, examined throughout the trial, and are integrated with the primary endpoints, such as survival, to examine the patient's progress and response to the trialled chemotherapy. With the support of a multidisciplinary cancer team, validated, cachexia‐specific quality‐of‐life tools would track a patient's cachexia trajectory during a trial and instigate supportive care when required. As trials are arduous, lengthy and accompanied by the patient's high symptom burden, incorporating these findings would promote robust trial design supporting the on time/on dose requirements and minimizing treatment toxicity. This would inevitably improve the survival and quality‐of‐life of patients undergoing gastrointestinal cancer chemotherapy trials and provide valuable insight of cachexia's assessment in clinical practice.

## Funding

No funding sources were utilized.

## Conflict of interest

The authors declare that they have no conflicts of interest.

## Data Availability

The data that support the findings are openly available on the respective clinical trials websites as referenced in this study. The data curation and analysis are available from the corresponding author upon reasonable request.
